# Kate Mountstephen

**Published:** 1887-09-03

**Authors:** C. G. Furley


### Kate Mountstephen.

By C. G. Furley.

(Continued from p. 370, vol. ii.)

### Chapter II.—"An Ounce of Practice!"

Kate Mountstephen had changed her habit for a dark velvet gown, and was seated before the fire reading. Her father, reassured by her appearance at the luncheon table, and happy in the knowledge that his darling was safe for this day at least, had gone out on a long round; while Kate consoled herself for the loss of a further run with the hounds by perusing Esmarch's work on field surgery in Princess Christian's translation. She had a pencil in her hand, with which she was marking passages which struck her as being of special importance, and occasionally entering an objection to the learned author's views.

"I suppose it is very unfeminine of me," she said to herself, pausing in her occupation, "to feel more interested in this book than in a novel. A woman is never expected to care for the mere physical frame, which, after all, sets the key of our emotions; and while she is supposed to be most desirous of helping her fellow-mortals there is a general impression—round Coteswold at least—that she shouldn't have any practical knowledge how to do it. Well, I must be content with the approval of my dear old father, my own conscience, and the poor folks whom I have helped in some degree to bear weakness and pain."

Putting aside reflections, she was about to resume her book, when a knock came to the door, and the old housekeeper put her head into the room.

"Oh! Miss Kate, I don't know what to do," she exclaimed.

"What has happened, Willis?" asked Kate.

"They've brought a poor gentleman here what's hurt himself out hunting. The friend who's with him says as he lives ten miles away; and the nearest doctor from this is four miles off; and the master's not in, and I don't know what to do. Should I tell him to come in and wait for master, Miss Kate?"

"Certainly. Take him and his friend into the consulting-room. Does he seem to be suffering much?"

"That he does. It's his arm that's injured, and you can see by his face that it is hurting him cruel bad."

"Well, bring him in; and I'll see if I can get anything to soothe the pain."

Kate waited a few minutes till the patient should be brought in, and then followed Mrs. Willis to the consulting-room. Harry Barnett was sitting on a couch near the fire, his white face and set teeth showing how keen was the pain he was suffering, while Captain Mitchell bent over him, and the old woman stood near with a glass of brandy, which she was offering to the patient.

"I am sorry my father is out," said Miss Mountstephen, as she approached. "Can you tell me what the injury is?"

"The huntsman said he thought the arm was dislocated," answered Mitchell; while Harry, unaccountably vexed at being seen by the young lady in such a plight, added: "You see, Miss Mountstephen, I did come to grief for lack of your guidance."

The ghost of a smile passed over Kate's face, which was paler than its wont. "I must try to repair the harm my absence has done," she said. "I can see that your arm is dislocated, and I think I can ease the pain if you will trust me."

"I would be grateful if you could lessen it in the slightest degree," he replied in a faltering voice.

She disappeared for a minute into the adjoining surgery and returned with some lint and bandages in her hands. Then she slit up the sleeve of his coat, so that it could be taken off easily, and made him lie face downwards on the couch.

"Can I help you in any way?" asked Mitchell.

"No," she answered; and observing the strained expression on his face, she added: "You are not accustomed to this sort of work; I think you had better go into the surgery there for a few minutes. Willis is used to such scenes, and knows how to give me all the help I require."

When Mitchell had withdrawn she cut up Barnett's clothing, till the shoulder, swollen and distorted, lay exposed to view.

"I must give you a few moments of intense pain," she said, "worse than what you are enduring now, but it will relieve you in the end."

"I will try to bear it as well as I can."

"No, don't. If you put any tension on your muscles you will make my task harder. Make no effort, and don't be ashamed to cry out."

He displayed no weakness, however, during the few seconds which it took her, by the utmost exertion of her strength, to put the arm into joint again. Then she deftly bandaged it up, and bidding Willis give the patient, who had almost fainted during the operation, some brandy, rested after her task, leaning against a table, breathless, and trembling a little.

"It has been too much for you, Miss Kate," said Willis.

"A little; I am not used to such tremendous pulling. I find there's a difference between a man's arm and that of a child. I shouldn't make a good surgeon."

While Miss Mountstephen was speaking Willis had raised Barnett up, and having draped him in the shawl which usually covered her own portly shoulders, was reviving him with occasional spoonfuls of the spirit. She had been with her young mistress in more than one emergency, when Kate had felt compelled to exercise her surgical skill, and while feeling the most fervent admiration for her, seconded her admirably in every point.

"Do you feel any pain elsewhere than in your shoulder?" asked Kate.

"Yes, every breath I draw hurts me. All my left side aches; but it is nothing to the pain in my shoulder."

"Ribs broken, probably," said Kate to herself. To-

Barnett she merely said, "You must wait and see my father. I have no doubt he will put you right. Meanwhile you must lie down. I know by experience what a terrible shock a fall gives one, and perfect rest is essential to get over it."

She gave a few directions to Willis, who left the room; then she called in Captain Mitchell.

"Your friend is considerably shaken," she said to the latter. "He will not be able to be removed to-night, possibly not for a few days; therefore I have ordered a room to be prepared for him, and I have no doubt you will help him to get to bed. Mrs. Willis will tell you when it is ready, and I shall be pleased if you will join me in the drawing-room afterwards. You will of course dine with us and stay overnight."

She left the room with an appearance of calmness she was far from feeling; for in spite of public rumour she was not in the habit of attempting surgical work except when, as in this case, a patient must endure hours of agony if she did not try to relieve it; and she felt more shaken at that moment than either those who admired or those who ridiculed her would readily have believed.

"Do you feel easier?" asked Mitchell of his friend.

"Much," answered Harry; then as an afterthought, "That girl is wonderful!" he said.

"Isn't she!" replied the other laconically.

"She must be very strong; but her touch is gentle and her voice very sweet."

"Yes; she is a thoroughly womanly woman." Then Willis returned, and Harry was led off to bed.

"Well, Kate, what have you been doing this time?" asked Dr. Mountstephen, when he entered the drawing-room a couple of hours later. He had seen Willis in the hall, and had learned what had occurred during the afternoon.

"I couldn't help it, papa," she answered, with a smile. "The poor fellow was suffering such pain that it would have been equally cruel to have let him be jolted four miles to reach Dr. Hayes, or to let him remain here without attempting to relieve him during all this time. I had to do what I could for him, and I think I managed the business quite right; but it was what you call 'a tough job,' and I don't want ever to try it again."

"Willis tells me you think he has a broken rib, too."

"I fear so; but you will find out."

"If that's the case we shall have him laid up here for some time."

"I suppose so."

"Then we had better send for your aunt Helen."

"As you like, papa," answered Kate, in a tone of meek deprecation. She did her best to like Mrs. Jeffreys, her aunt Helen, because she was her dead mother's sister, but found the task a little difficult. Mrs. Jeffreys disapproved entirely of her niece doing anything more practical for her poorer neighbours than giving them an occasional tract; and being one of those people who have an exaggerated fear of infection, she always made it a condition of visiting Coteswold that Kate should not go into one of "those dreadful cottages" during her stay, as she felt sure that the inmates of them were all more or less ill.

"But, aunt," Kate had protested once, "papa is every whit as likely to bring infection as I, and you don't want him to leave off visiting his patients."

"Certainly not, Kate. That is his duty, and I would not stand between any man and the performance of his duty."

"But that is not the point. Whether or not it is his duty to visit sick people, and whether or not it isn't mine, the liability to infection is the same."

"Not at all," said Mrs. Jeffreys, severely. "Providence watches over people who are doing the work it has given them to do, but not over those who are intruding in places they should never enter, and who are neglecting the social duties belonging to their position. I hope you don't deny the existence of Providence, Kate."

Kate failed to perceive the aptness and force of the argument; but she found protest useless, and submitted to aunt Helen's short and infrequent visits as patiently as she could.

"She will be more shocked than ever this time," she said to herself now. "Spending the night with a child who has bronchitis is nothing to setting a man's shoulder and having one's patient in the house. Oh! what lectures on my eccentricity I shall get within the next week."

But for once Miss Mountstephen's prediction was unfulfilled. Mrs. Jeffreys did indeed call her a foolish child, but in the most amiable of tones, and modified by a congratulation on her "nonsensical studies" having proved really useful at last. Kate saw no greater usefulness in setting Harry Barnett's arm than in setting one of the village children's; but she was too glad to escape the anticipated scolding, to analyse her aunt's words very closely.

She would, however, have been surprised to hear the effusive way in which Mrs. Jeffreys spoke of her to Mr. Barnett, when she visited him in his room to see that he was quite comfortable. Assuredly Kate would have thought aunt Helen had taken leave of her senses, had she overheard her speaking in this fashion:

"Though I say it, who am her aunt, Mr. Barnett, you might have fallen into worse hands than my niece's, when you met with those terrible injuries."

"The injuries weren't so very deadly. I daresay I made more fuss about them than they deserved," answered Harry, ashamed—after the manner of his countrymen—that he had ever confessed to feeling pain; "but," he added fervently,—"but Miss Mountstephen is an angel, or better. I doubt if an angel could have set my shoulder."

"A true woman is better than an angel, at least to help a suffering or struggling man," observed Mrs. Jeffreys, sententiously, adding, after a pause—"dear Kate!"

"You know," said Harry, repentantly, after a few minutes' silence, "I always thought that a girl who went in for medicine, or anything like that, must become rather rough and abrupt in manner; and she isn't; she is perfectly sweet and womanly."

"Kate? Oh yes! I hope that *my* niece would never be guilty of anything unfeminine," replied Mrs. Jeffreys, with all the dignity of aunthood. "Had I thought that these charitable works of hers were likely in any degree to unsex her, I would have used all my influence to prevent her going on with them. But on the contrary, I saw that they helped in the development of a noble character. Woman's noblest mission is to aid the suffering."

These beautifully turned sentences impressed Harry, but, perhaps, not altogether as the speaker would have desired. "The old lady speaks like a copy-book," he confided to himself; but he admired the object of her praise too much to cavil greatly at her expressions. No doubt she was old-fashioned and formal, but in that case, thought unsuspecting Harry, it proved Kate's sweetness of character all the more that Mrs. Jeffreys approved of her undertaking tasks which in her own young days would have been deemed extremely unfeminine.

"Really," he said, making a comparison unconsciously, "a girl is quite as womanly in the hunting-field or the sick-room as at a ball or a garden-party; and it's so much nobler—at least the sick-room—I don't believe a lady really likes running a fox down, though she may care for the excitement of riding."

"Just so," said Mrs. Jeffreys with an inward smile. She knew what her niece was wont to say of her favourite amusement.

"Are your pillows quite comfortable?" she asked, by way of changing the key; and being assured that they were, she glided into another subject.

"You aren't quite a stranger to me, Mr. Barnett; I think I have met you at your uncle's house. I knew Mrs. Barnett before her marriage."

"I am sorry I did not recognise you; but I don't often go to my uncle's when they have people there, except now and again to a big crush."

"It was at a 'crush'—a very large reception—that you were pointed out to me."

"It's that 'pointing out' I hate," explained Harry. "Uncle Will's friends seem always to assume that I feel aggrieved by his marriage, and point me out to other people in a 'poor fellow,' pitying sort of way. And you know I don't feel like that at all. I don't deny that it made a difference to me in some ways, and some people were cooler after it, but really I can't see how my uncle wronged me in pleasing himself, and I like Mrs. Barnett very much. As for the baby, he is a splendid little fellow. Don't you think so?"

"He was a very fine child," answered Mrs. Jeffreys, with a slightly bewildered air. "Surely he can't know what has happened," she said to herself.

"Have you seen them lately?" she asked aloud.

"About ten days ago, just before I came here."

"Have you been here so long as that?"

"Yes; I came down to have a fortnight's hunting with Captain Mitchell, who lives about ten miles from here; but the first week we never got out, owing to that confounded frost—oh! I beg your pardon, Mrs. Jeffreys; but to have one's hardly-won holiday spoiled first in that way, and then by this idiotic spill I went in for yesterday, doesn't tend to improve one's temper."

"I am sure it does not. And it has been a sad frost. There have been several accidents."

"Yes, I suppose so; people will begin skating before the ice is strong enough."

"Evidently he doesn't know what has happened," again said the lady to herself; and she asked herself if she ought to tell him.

Before she could settle that point with her conscience Captain Mitchell was announced. He had gone home that morning, and now returned late in the afternoon to inquire for his friend, and to bring him some letters that had come that morning. Perhaps also to see Kate Mountstephen again; for the Captain still hoped—though in a desponding fashion, not much removed from despair—that she would reconsider her decision, and accept him as her husband. Poor Captain Mitchell! He did not know that he had himself brought a rival on the scene, and spoiled his last chance. Pity is akin to love in women's minds, above all when pity has been followed by practical help. A woman feels a sort of proprietorship in the man for whom she has done something—performed some task, given some aid, made some sacrifice—which, by the fine inscrutable logic of the feminine soul, changes itself into the belief that he has a certain proprietorship in her—the right to claim that help in all the emergencies of life, and to assure his claim by the bond which alone can make it ultimately possible.

"Why, who can this black-edged communication be from?" exclaimed Harry, turning over in his hands one of the three envelopes his friend had brought. "It has been sent to the office, I see, and then forwarded. I ought to have had it two days ago. I wonder what it's about. I don't know the writing."

"Better open it," advised Mitchell, laconically.

Mrs. Jeffreys, who had gone towards the door, meaning to leave the two men alone, paused, and looked back while Barnett opened the letter. As he glanced at the letter Harry uttered a brief exclamation of grief and horror.

"What is it?" exclaimed both his companions.

Harry extended the letter towards Mitchell, who read aloud the following note:—

"Dear Sir,—I am requested by Mrs. Barnett, who is at present overcome with grief, to inform you of the sad bereavement that has befallen her and her husband. Their infant son was out with his nurse this morning, when the girl, trying to cross a crowded street with the child in her arms, fell on the slippery ground in front of an omnibus. The nurse has escaped with a broken leg and some severe contusions, but the child received a wound on the head from the hoofs of one of the horses, which, I regret to say, has caused fracture of the skull with fatal results. The sudden and terrible death of his son has caused a great shock to your uncle's health, and Mrs. Barnett will be greatly obliged by your coming to see him as soon as possible, as both she and I hope that your presence may rouse him from the stupor into which he had fallen.—Faithfully yours,

"Charles Stephens, M.D."

"I must go at once," exclaimed Harry. "My poor old uncle—what a terrible blow!"

"But you can't go," exclaimed both his listeners. He protested; but the utmost concession he could obtain was a promise that the matter should be referred to Dr. Mountstephen. The doctor would not listen to the idea of Barnett's leaving.

"Travel to London in January with two ribs newly put up, an arm not yet quite settled in its old position, and a temperature at 100°. Certainly not! I won't allow it, and neither will Kate. We don't like to have our work spoiled by subsequent neglect or mismanagement. I will write to Dr. Stephens and explain why you can't come."

And after thinking it over Harry was quite content to remain where he was.

(*To be continued.*)

